# Effects of Severe Varus Deformity on Soft Tissue Balancing in Total Knee Arthroplasty

**DOI:** 10.3390/jcm12010263

**Published:** 2022-12-29

**Authors:** Il-Hoon Kwak, Sung-Sahn Lee, Jeounghun Lee, Dae-Hee Lee

**Affiliations:** 1 Department of Orthopaedic Surgery, Samsung Medical Center, Sungkyunkwan University School of Medicine, Seoul 06351, Republic of Korea; 2 Department of Orthopaedic Surgery, Ilsan Paik Hospital, Inje University School of Medicine, Goyang-si 10380, Republic of Korea

**Keywords:** total knee arthroplasty, soft tissue balancing, gap difference, hip–knee–ankle varus angle, joint line convergence angle

## Abstract

This study aimed to establish the effect of severe varus deformity on soft tissue balance in total knee arthroplasty (TKA), which is not yet well established. We retrospectively enrolled 205 patients (270 knees) who underwent primary TKA using the measured resection technique. Four intraoperatively measured TKA gaps and gap differences were compared between the severe varus deformity group (Hip-knee-ankle [HKA] varus angle ≥ 10°) and the mild varus deformity group (HKA varus angle < 10°). Pearson’s correlation analysis and multiple linear regression analysis were used to investigate the factors affecting flexion and extension gap differences (FGD and EGD). A receiver operating characteristic curve was applied to assess the cut-off value of the HKA varus angle to discriminate the rectangular gap. The FGD (1.42 ± 1.35 mm vs. 1.05 ± 1.16 mm, *p* = 0.019) and the EGD (1.45 ± 1.32 mm vs. 0.97 ± 1.53 mm, *p* = 0.006) were significantly larger in severe varus deformity group than in mild varus deformity group. The HKA varus angle was positively correlated with both FGD (r = 0.264, *p* < 0.001) and EGD (r = 0.319, *p* < 0.001) and was an influencing factor for FGD (β = 0.232, *p* = 0.040) and EGD (β = 0.229, *p* = 0.037). A preoperative HKA angle of 12.4° was selected as the cutoff value to discriminate between rectangular and trapezoidal gaps. Thus, the severity of varus deformity (HKA varus angle) was found to be a significant factor affecting the mediolateral gap difference in TKA. When performing TKA in a knee with an HKA varus angle ≥ 12.4°, a trapezoidal gap is more likely to be expected. Level of evidence III, case–control study.

## 1. Introduction

Total knee arthroplasty (TKA) is the most reliable surgery to relieve pain and disability caused by advanced arthritis of the knee in elderly patients [1,2]. The importance of soft tissue balancing in TKA has been emphasized by several authors [3]. For long-term successful TKA, equalizing the rectangular flexion and extension gaps is crucial [4,5,6,7,8].

Varus knee deformity is the most common deformity in patients who undergo primary TKA. Osteoarthritic varus knees are characterized by cartilage and bone loss in the medial compartment of the knee joint as well as medial contracture and lateral laxity of ligaments and soft tissues [9]. Based on our experience and literature review, soft tissue balancing in TKA is more challenging in patients with severe varus knee deformity as compared to those with mild varus deformity [10]. However, the effects of varus deformity severity on soft tissue balancing in TKA are not well established yet. Furthermore, it is difficult to exactly quantify soft tissue balancing intraoperatively.

This study aimed to investigate the effect of preoperative varus deformity severity on soft tissue balancing. It was hypothesized that patients with severe varus deformity are more likely to have a poorly balanced gap with a larger mediolateral gap difference compared to patients with mild varus deformity. 

## 2. Materials and Methods

### 2.1. Study Design and Patients

This study is a retrospective study of the prospectively collected TKA dataset in our institution. This study included 205 patients (270 knees) who underwent primary TKA with posterior cruciate ligament substituting (PS) implants (Attune system, DePuy Synthes, West Chester, PA, USA; Persona system, Zimmer Biomet, Warsaw, IN, USA) between December 2018 and May 2021. The inclusion criteria were patients with substantial pain and loss of function due to advanced osteoarthritis of the knee with varus knee alignment. The exclusion criteria were patients who underwent TKA revision surgery (17 cases) and previous knee surgery requiring the removal of metallic implants (1 case) and those with genu valgum deformity (18 knees). All surgeries were performed by a single experienced surgeon. We divided the 270 knees into two groups based on the magnitude of the preoperative hip–knee–ankle (HKA) varus angle. Severe varus deformity was defined as an HKA varus angle greater than 10°, whereas mild varus deformity was defined as an HKA varus angle less than 10° [11,12,13]. The following data were compared between the two groups: age, sex, height, weight, body mass index (BMI), preoperative knee range of motion (ROM), and Hospital for Special Surgery (HSS) scores. The study design was approved by our institutional review board (SMC 2022-09-137), and informed consent was obtained from each patient.

### 2.2. Surgical Technique and Gap Measurement

**Figure 1:**
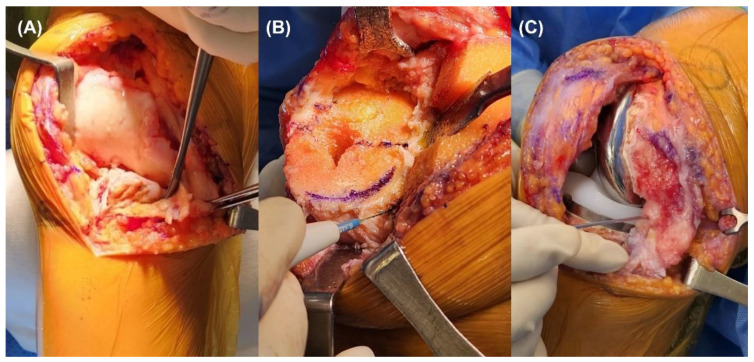
Sequential medial soft tissue release. (**A**) Deep medial collateral ligament release was routinely performed. When medial soft tissue is still tight after meticulous osteophyte removal and bone cutting, (**B**) releasing of tibial insertion of the semimembranosus was performed. (**C**) Pie-crusting technique for medial collateral ligament using 18-gauge needle was performed as the previous method.

All total knee replacement arthroplasties were performed using the measured resection technique. After a midline skin incision and medial parapatellar approach, the deep medial collateral ligament was preliminarily released. Both cruciate ligaments were resected for using PS-type implants. The osteophytes were meticulously removed prior to bone cutting. For the distal femur cut, the valgus cutting angle was set perpendicular to the mechanical axis, which was measured on a preoperative radiograph of the lower extremity. Proximal tibial cutting was performed perpendicular to the mechanical axis using an intramedullary guide. A femoral anteroposterior cut was performed using three landmarks: trans-epicondylar, posterior condylar, and anteroposterior axes. After completion of bone cutting, additional medial soft tissue release was performed if medial tightness remains after component trial implantation if the medial tightness remained. This included the partial release of the tibial insertion of the semimembranosus and medial collateral ligament pie-crusting (Figure 1). The medial release was performed carefully to prevent laxity of the medial side soft tissue. In order to avoid medial instability and extensive joint line elevation, lateral laxity within 3 mm was allowed as in previous studies [14,15].

**Figure 2:**
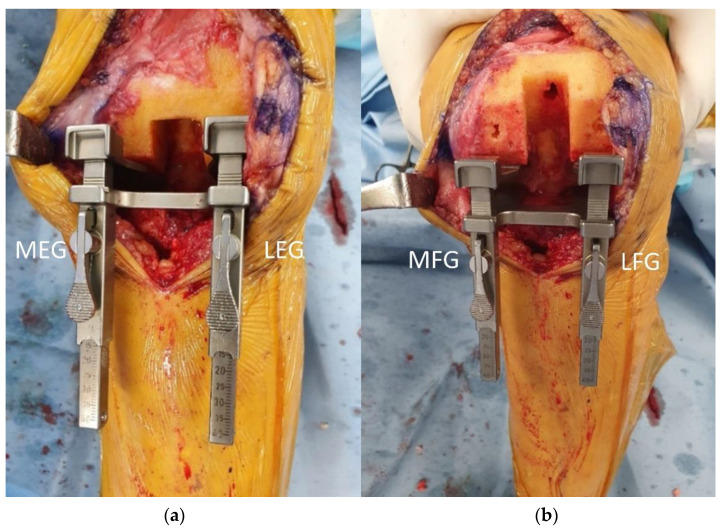
Measuring four total knee arthroplasty gaps (MFG, LFG, MEG, LEG) using a device similar to a lamina spreader and a tensor with a slide ruler. (**a**) Measurement of extension gaps (MEG, LEG). (**b**) Measurement of flexion gaps (MFG, LFG); MEG: medial extension gap; LEG: lateral extension gap; MFG: medial flexion gap; LFG: lateral flexion gap.

The four TKA gaps, including medial extension gap (MEG), lateral extension gap (LEG), medial flexion gap (MFG), and lateral flexion gap (LFG), were measured using a gap measuring device to quantify soft tissue balancing (Figure 2). Medial and lateral gaps were measured at full knee extension and 90° flexion, respectively. A distraction force of 40 lb (18.7 kg) was applied to the measuring device which was lamina spreader and tensor with a side ruler (B. Braun Aesculap, Tuttlingen, Germany) [3,9,16,17,18,19,20]. The measurements were repeated twice and the mean value was recorded. Flexion gap difference (FGD) was defined as the subtraction of MFG from LFG (LFG-MFG), and extension gap difference (EGD) was defined as the subtraction of MEG from LEG (LEG-MEG). The trapezoidal gap was defined as a TKA gap measurement that showed at least one gap difference greater than 3 mm. Otherwise, the gap was defined as rectangular [18]. 

### 2.3. Clinical and Radiologic Assessments

Preoperative HSS scores and ROM were measured on the day of admission. Postoperative HSS scores and ROM were recorded at the outpatient clinic at 3, 6, 9, 12, and 24 months after surgery.

**Figure 3:**
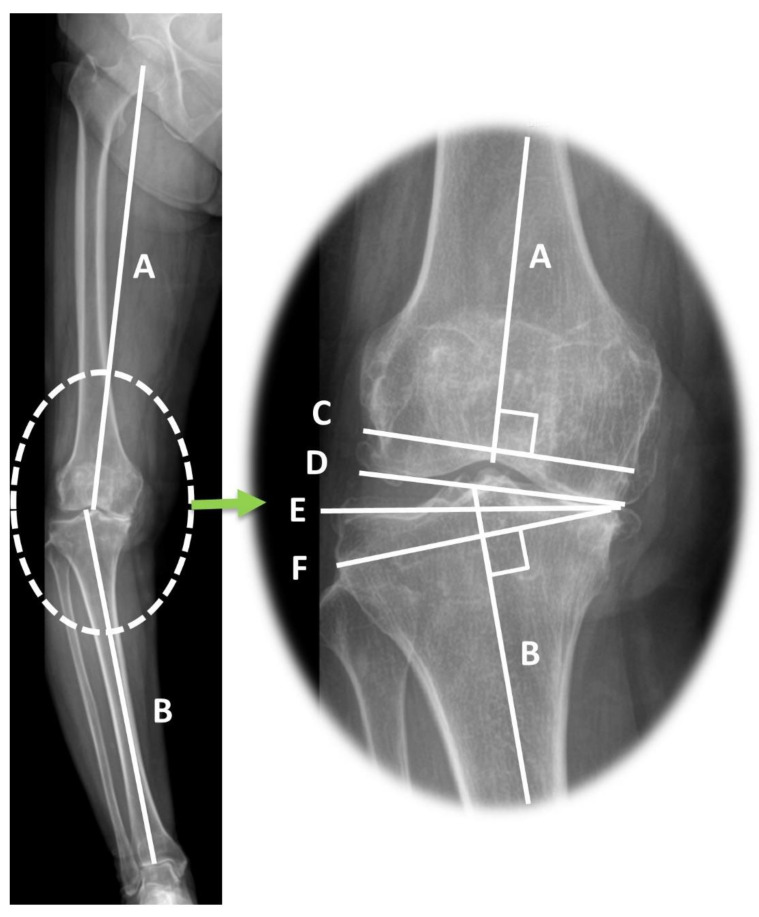
Limb coronal alignments and bone cutting lines demonstrated in standing long leg radiograph. A: Mechanical axis of the femur, B: Mechanical axis of the tibia, C: Distal femur cutting line, D: Distal femoral articular surface, E: Proximal tibial articular surface, F: Proximal tibial cutting line. HKA varus angle: acute angle between A and B; mLDFA: lateral angle between A and D; MPTA: medial angle between B and E; JLCA: angle between D and E. The distal femur (line C) and proximal tibia cutting (line F) were perpendicular to the mechanical axis of the femur (line A) and the mechanical axis of the tibia (line B). The gap between lines C and F (extension gap) shows a trapezoidal gap after bone cutting.

**Figure 4:**
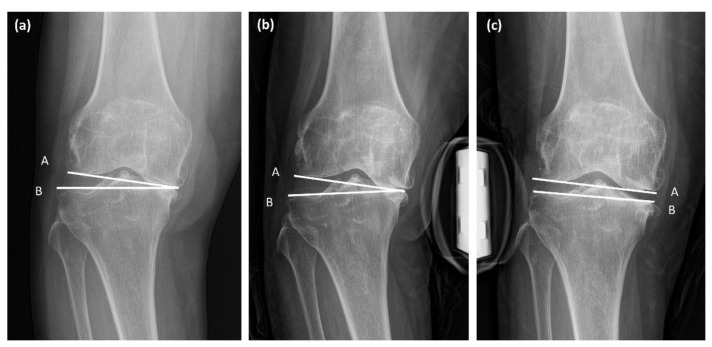
Measurement of joint line convergence angle (JLCA) and varus/valgus stress JLCA. Line A and B is the line connecting the articular surface of the distal femur and proximal tibia. (**a**) JLCA standing; (**b**) JLCA under varus stress; (**c**) JLCA under valgus stress.

Preoperative radiographic parameters representing coronal alignment of the lower extremity were measured using a measurement tool in the picture archiving system software (Centricity Enterprise Web; 2006 GE healthcare, Chicago, IL, USA). The HKA varus angle was defined as the acute angle between the mechanical axes of the femur and tibia, which are the lines connecting the centers of the hip, knee, and ankle [21,22]. The mechanical lateral distal femoral angle (mLDFA) was measured as the lateral angle between the mechanical axis of the femur and the distal femoral joint line. The medial proximal tibial angle (MPTA) was defined as the medial angle between the mechanical axis of the tibia and the tangent to the tibial plateau line [9] (Figure 3). The joint line convergence angle (JLCA) and JLCA under varus/valgus stress were measured on standing anteroposterior and stress radiographs (Figure 4). JLCA was defined as the angle between the line connecting the articular surfaces of the distal femur and the proximal tibia [19]. The medial JLCA apex (varus) was assessed as positive, while the lateral JLCA apex (valgus) was measured as negative [23]. JLCA was also measured on the stress radiograph, which was taken under varus or valgus stress of 150 N to the knee in extension using a Telos device (Fa Telos, Medizinisch-Technische, Greisheim, Germany). All radiographic values were measured twice by two orthopedic surgeons with an interval of 2 weeks between measurements. 

### 2.4. Statistical Analyses

Priori power analysis was performed at an α level of 0.05 and a power of 0.80 (Medcalc version 19.0.7, MedCalc Software Ltd., Ostend, Belgium) to determine the sample size to detect even a 1 mm difference of EGD between the severe varus deformity and mild varus deformity groups. The means and standard deviations were obtained from the pilot study.

The reliability of the measurements of preoperative coronal alignment angles was determined by calculating the intraclass correlation coefficient, which was used to quantify the inter-observer and intra-observer measurement variability.

The intraoperative gap measurements and preoperative radiologic parameters were compared between groups with an HKA varus angle ≥ 10° and <10° using Student’s t and Mann–Whitney U tests. The correlation coefficients between the gap differences and radiologic parameters were analyzed using Pearson’s correlation analysis. Multiple linear regression analysis was used to identify the independent variables that affected gap differences. The postoperative ROM and HSS at the latest follow-up visit were compared between the two groups using a t-test. Statistical analyses were performed using IBM SPSS Statistics version 27 (IBM Corporation, Armonk, NY, USA). All data are presented as mean and standard deviation. Statistical significance was set at *p*-value < 0.05.

Receiver operating characteristic (ROC) analysis (MedCalc version 19.0.7, MedCalc Software Ltd., Ostend, Belgium) was used to evaluate the cutoff value of preoperative HKA that differentiates rectangular and trapezoidal gaps. 

## 3. Results

Based on the results of the power analysis, 40 and 54 subjects were estimated to have a 1 mm difference in EGD between the severe varus deformity and mild varus deformity groups, respectively. The current study included 116 and 154 knees in each group, respectively, indicating an adequate power (0.886). The inter-observer and intra-observer reliabilities of the preoperative coronal alignment angles ranged from 0.822 to 0.881, indicating good reliability.

Depending on the preoperative HKA varus angle, 154 knees were included in the severe varus deformity group (HKA varus angle ≥ 10°) and 116 knees were included in the mild varus deformity group (HKA varus angle < 10°). No significant differences were observed in age, sex, height, weight, BMI, preoperative knee ROM, or HSS scores (Table 1).

All mean intraoperative TKA gaps were larger in the severe varus deformity group than those in the mild varus deformity group. The FGD and the EGD were also significantly larger in the severe varus deformity group than those in the mild varus deformity group (Table 2). 

The preoperative measurements of the HKA varus angle, mLDFA, JLCA, and JLCA under varus stress were larger in the severe varus deformity group than those in the mild varus deformity group. While MPTA was smaller in the severe varus deformity group (Table 3).

Pearson’s correlation test results showed that FGD was positively correlated with preoperative HKA varus angle, mLDFA, and JLCA under varus stress and negatively correlated with MPTA. EGD was positively correlated with HKA varus angle, JLCA, and JLCA under varus stress and negatively correlated with MPTA (Table 4).

Multiple linear regression analysis showed that the preoperative HKA varus angle and JLCA under varus stress were predictors of FGD, while preoperative HKA varus angle, MPTA, and JLCA under varus stress were factors affecting EGD (Table 5).

**Figure 5:**
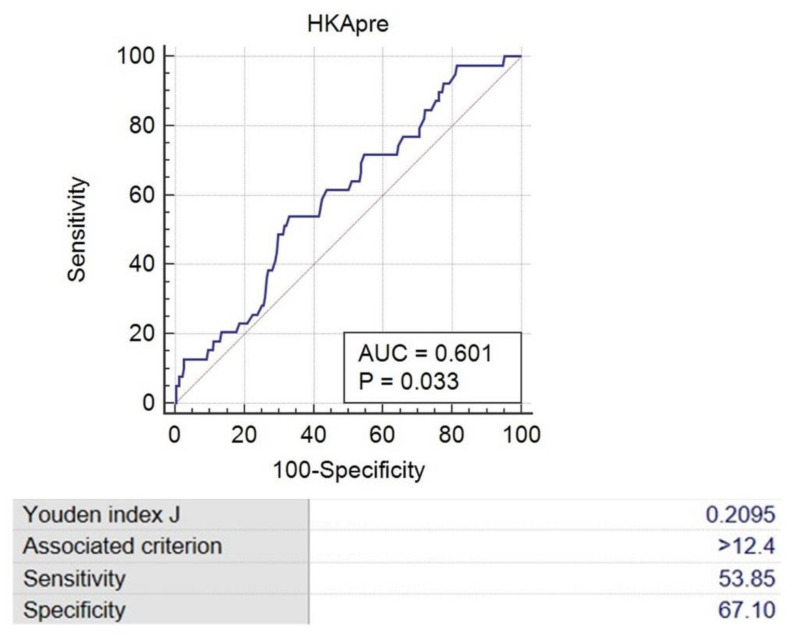
Receiver operating characteristic curve to evaluate cutoff value of preoperative HKA that discriminates rectangular and trapezoidal gap.

A ROC analysis was performed to determine threshold values for the HKA varus angle to define the trapezoidal group (trapezoidal gap > 3 mm with at least one of the FGD and EGD). The area under the curve (AUC) for the HKA varus angle in the trapezoidal group was 0.601 (95% confidence interval [CI], 0.540–0.660). An HKA varus angle of 12.4° was found to be the cutoff value for the trapezoidal group, with a sensitivity of 53.85% and specificity of 67.10% (Figure 5).

Postoperative clinical outcomes, including knee joint ROM (119.1 ± 7.5° vs. 117.0 ± 11.0°, *p* = 0.078) and postoperative HSS score (81.4 ± 11.9 vs. 80.8 ± 13.7°, *p* = 0.742) showed no statistical difference between the severe varus deformity and mild varus deformity groups at the latest follow-up.

## 4. Discussion

The present study showed that preoperative knee joint alignment affects the soft tissue balance in TKA. Severe varus deformity resulted in an increase in both FGD and EGD. Furthermore, knee lateral soft tissue laxity (JLCA under varus stress) is a factor that increases the mediolateral gap differences.

Conventionally, rectangular soft tissue balance is important to a successful TKA [24]. Therefore, medial soft tissues are frequently released to achieve proper soft tissue balance in case of varus deformity [25]. There are concerns about the adverse effects associated with excessive MCL release [26,27,28]. Extensive MCL release may be associated with mid-flexion instability and abnormal anterior motion of the femur in deep knee flexion. Medial instability after TKA may be the causative factor for postoperative knee pain and poor functional outcomes [29]. Due to the disadvantages of excessive MCL release, some studies had investigated the influence of medial tight trapezoidal gap balance on postoperative outcomes. Lateral soft tissue is looser than medial soft tissue in normal knee kinematics [30]. Sekiya et al. reported that the residual lateral ligamentous laxity observed in varus deformity was spontaneously corrected after TKA with neural alignment [14]. Recently, TKA with a medial stabilizing technique, which minimizes medial soft tissue release and accepts trapezoidal gap, was introduced and showed similar postoperative results compared with conventional rectangular gap-balanced TKA [15]. We think that it is best to obtain a rectangular gap without extensive medial release, however in situations where excessive medial release is required, it is better to allow a little lateral laxity than to obtain a rectangular gap with the risk of medial instability and excessive joint line elevation.

Recently, kinematic-aligned TKA was introduced and performed. This technique was based on the theory to preserve normal knee kinematics and to minimize soft tissue release based on pre-arthritic joint status [31]. The patients’ function and satisfaction were dependent on preserving pre-arthritic kinematics for providing neurosensory feedback [32]. Many studies have investigated the effect of kinematic-aligned TKA on clinical outcomes, which were associated with similar or better clinical outcomes in short-term studies [31]. Kinematic aligned TKA employed femoral resections placed in a more valgus position and tibial resections placed in a more varus position compared to mechanical aligned TKA. Moreover, need for soft tissue release was significantly less in kinematic-aligned TKA [33]. We think kinematic-aligned TKA could be an alternative option in patients who had preoperative severe varus deformity. 

The primary cause of mediolateral gap differences (trapezoidal gap) in severe varus knees is the principle of mechanically aligned bone cutting. In mechanically aligned TKA, the distal femur and proximal tibia are resected at a right angle (90°) to their respective mechanical axes [34,35]. The angle between the mechanical axis and bone cutting line and the thickness of the distal femur and proximal tibia bone cutting is shown in Figure 3. Lines C and F indicate bone-cutting lines that are perpendicular to their respective mechanical axes (lines A and B). The severity of the varus deformity of the proximal tibia and distal femur is directly proportional to the angle between tangential lines of the distal femur and proximal tibia articular surface and osteotomy line (∠C,D and ∠E,F, Figure 3). As a result of mechanically aligned bone cutting on varus-deformed knees, a larger thickness of bone is inevitably cut in the lateral compartment [36,37]. The discrepancy in bone resection between the medial and lateral sides is more prominent in the proximal tibia than in the distal femur. Every osteoarthritic knee with varus alignment shows proximal tibia varus, but distal femur varus is not always observed [23]. In addition, in the present study, the proximal tibia showed a greater varus magnitude than the distal femur (mean MPTA 84.3° and mLDFA 89.5°). For this reason, trapezoidal flexion and extension gaps are more likely to originate from proximal tibia varus deformity than from distal femur varus deformity. This finding also supports the result of the present study that MPTA was correlated with FGD and EGD and was an influencing factor for EGD. 

The lateral soft tissue laxity of the knee also contributes to mediolateral gap differences during TKA. Knee osteoarthritis with severe varus deformity is associated with tightened medial soft tissue structures and lax lateral structures [38]. In the present study, we assessed medial and lateral soft tissue tension using the JLCA on standing anteroposterior and varus/valgus stress radiographs (Figure 3). The results demonstrated that lateral ligament laxity (JLCA under varus stress) was significantly larger in the severe varus deformity group than in the mild varus deformity group. This was determined to be an influencing factor for both FGD and EGD. However, the medial ligament laxity (JLCA under valgus stress) showed no statistical difference between the severe and mild varus deformity groups. The pathogenesis of lateral soft tissue laxity in varus osteoarthritic knees is believed to be related to the adduction moment of the knee [37,39]. In osteoarthritic varus knees, the ground reaction force passes far medially to the center of the knee and produces the knee adduction force [19]. Weight-bearing as a part of daily activities increases the adduction moment and aggravates the laxity of the lateral soft tissues [37].

Our study suggests that the possibility of a large mediolateral gap difference (trapezoidal gap) can be predicted preoperatively by measuring coronal alignment. The analysis of the ROC curve in this study identified a cutoff point value of 12.4° for the HKA varus angle to discriminate between rectangular and trapezoidal gaps. This finding could indicate that when performing TKA in patients with an HKA angle ≥ 12.4°, a higher possibility of trapezoidal gap formation can be expected; therefore, the surgeon should pay more attention to achieving acceptable soft tissue balancing with rectangular flexion and extension gap during TKA. In addition, this result also suggests that some magnitude of mediolateral gap differences could be acceptable in patients with severe varus knees. 

The present study had several limitations. First, the follow-up period was relatively short. The duration of the study ranged from 1.5 to 4 years, and no loosening or failure of the components was reported. The long-term effects of trapezoidal gaps on the prognosis of TKA, including implant survivorship and clinical outcomes, need to be analyzed in future studies. Second, every surgery in the study was performed using PS-type implants. PS-type TKA is preferred because it is reproducible in most surgical situations without concern for PCL tension, especially in severe varus deformity [40]. Since previous studies have revealed that PCL release has a significant effect on the flexion gap [3,6], this study may not be applicable to PCL-retaining implants. Third, TKAs were performed for conventional alignment in the current study. Kinematic-aligned TKAs are difficult to apply to our results. Lastly, due to anesthesia and the relaxation of muscles, the effects of dynamic stabilizers in TKA gaps could not be considered. The effects of patellar eversion and extensor mechanisms on the gaps may differ individually [6]. 

## 5. Conclusions

The medial and lateral flexion and extension gaps (MFG, LFG, MEG, and LEG) were larger in the severe varus deformity group (HKA varus angle ≥ 10°) than in the mild varus deformity group (HKA varus angle < 10°). The magnitude of preoperative varus deformity (HKA angle) was the factor affecting the increase in the mediolateral FGD and EGD. Patients with an HKA varus angle > 12.4° were more likely to have a poorly balanced gap or trapezoidal knee with soft tissue imbalance after TKA.

## Figures and Tables

**Table 1:** Demographic characteristics of subjects classified as severe varus deformity group (hip–knee–ankle [HKA] varus angle ≥ 10°) and mild varus deformity group (HKA varus angle < 10°).

	Overall	Varus < 10°	Varus ≥ 10°	*p*-Value
Sample size (number)	270	116	154	
Sex (male/female)	52/218	18/98	34/120	0.176
Age (years)	72.3 ± 6.1	71.4 ± 6.0	72.9 ± 6.2	0.41
Height (cm)	154.3 ± 7.3	155.5 ± 7.4	153.4 ± 7.2	0.553
Weight (kg)	65.0 ± 11.7	66.9 ± 12.4	63.5 ± 10.9	0.067
Body mass index (kg/m^2^)	27.2 ± 3.8	27.6 ± 4.0	26.8 ± 3.7	0.135
Preoperative ROM	110.4 ± 15.6	112.6 ± 15.8	108.7 ± 15.3	0.438
Preoperative HSS	23.3 ± 12.6	24.6 ± 13.0	21.9 ± 11.6	0.603

Results are reported as mean ± standard deviation unless otherwise indicated. ROM: range of motion; HSS: Hospital for Special Surgery.

**Table 2:** Comparison of total knee arthroplasty gaps between severe varus deformity group (hip–knee–ankle [HKA] varus angle ≥ 10°) and mild varus deformity group (HKA varus angle < 10°).

	Overall	Varus < 10°	Varus ≥ 10°	*p*-Value
	(N = 270)	(N = 116)	(N = 154)	
MFG (mm)	19.20 ± 1.99	18.72 ± 1.68	19.57 ± 2.13	<0.001
LFG (mm)	20.47 ± 2.13	19.76 ± 1.84	20.99 ± 2.19	<0.001
MEG (mm)	19.11 ± 2.05	18.67 ± 1.78	19.44 ± 2.18	0.002
LEG (mm)	20.35 ± 2.28	19.64 ± 1.98	20.89 ± 2.35	<0.001
FGD(LFG-MFG) (mm)	1.26 ± 1.28	1.05 ± 1.16	1.42 ± 1.35	0.019
EGD(LEG-MEG) (mm)	1.24 ± 1.43	0.97 ± 1.53	1.45 ± 1.32	0.006

Results are reported as mean ± standard deviation unless otherwise indicated. Boldface text indicates a parameter that differed significantly between the two groups (*p* < 0.05). MFG: medial flexion gap; LFG: lateral flexion gap; MEG: medial extension gap; LEG: lateral extension gap; FGD: flexion gap difference; EGD: extension gap difference.

**Table 3:** Radiologic measurement parameters in the severe varus deformity group (hip–knee–ankle [HKA] varus angle ≥ 10°) and mild varus deformity group (HKA varus angle < 10°).

	Overall	Varus < 10°	Varus ≥ 10°	*p*-Value
	(N = 270)	(N = 116)	(N = 154)	
HKA varus angle (°)	11.07 ± 4.91	6.73 ± 2.27	14.33 ± 3.66	<0.001
mLDFA (°)	89.81 ± 2.41	89.03 ± 2.33	90.77 ± 5.06	0.001
MPTA (°)	84.31 ± 3.02	85.86 ± 2.40	83.14 ± 2.92	<0.001
JLCA (°)	5.35 ± 2.48	4.16 ± 1.99	6.24 ± 2.44	<0.001
JLCA under varus stress (°)	7.32 ± 2.80	6.88 ± 2.13	8.01 ± 3.03	0.001
JLCA under valgus stress (°)	2.28 ± 1.79	−0.12 ± 2.31	−0.05 ± 2.30	0.805

Results are reported as mean ± standard deviation unless otherwise indicated. Boldface text indicates a parameter that differed significantly between the two groups (*p* < 0.05). HKA: Hip-knee-ankle; mLDFA: mechanical lateral distal femoral angle; MPTA: medial proximal tibia angle; JLCA: joint line convergence angle.

**Table 4:** Correlations between gap differences and radiologic measurement parameters.

	FGD	EGD
HKA varus angle (°)	0.264 (<0.001)	0.319 (<0.001)
mLDFA (°)	0.123 (0.043)	0.089 (0.146)
MPTA (°)	−0.192 (0.002)	−0.323 (<0.001)
JLCA (°)	0.105 (0.085)	0.140 (0.021)
JLCA under varus stress (°)	0.153 (0.021)	0.177 (0.004)
JLCA under valgus stress (°)	−0.097 (0.113)	0.014 (0.814)

Results are reported as Pearson correlation coefficient (*p*-value). Boldface text indicates a parameter that differed significantly between the two groups (*p* < 0.05). MPTA: medial proximal tibial angle; mLDFA: mechanical lateral distal femoral angle; JLCA: joint line convergence angle; FGD: flexion gap difference; EGD: extension gap difference.

**Table 5:** Multiple regression analysis of factors that affect gap differences.

Dependent Variables	Independent Variables	Non-Standardized Coefficients		Standardized Coefficients	
		B	SE	β	*p*-Value
FGD	HKA varus angle	0.06	0.029	0.232	0.04
	MPTA	−0.046	0.038	−0.11	0.239
	mLDFA	0	0.042	0.153	0.639
	JLCA under varus stress	0.072	0.028	0	0.012
	JLCA under valgus stress	−0.102	0.092	−0.103	0.092
EGD	HKA varus angle	0.066	0.031	0.229	0.037
	MPTA	−0.114	0.041	−0.244	0.006
	mLDFA	−0.025	0.045	−0.042	0.58
	JLCA under varus stress	0.093	0.031	0.177	0.004
	JLCA under valgus stress	−0.005	0.04	−0.008	0.894

B: unstandardized coefficients; SE: standard error; β: standardized coefficients; FGD: flexion gap difference; EGD: extension gap difference; HKA: hip knee ankle; MPTA: medial proximal tibial angle; mLDFA: mechanical lateral distal femoral angle; JLCA: joint line convergence angle.

## Data Availability

Not applicable.
